# Characteristics and Behaviors of Anonymous Users of Dark Web Platforms Suspected of Child Sexual Offenses

**DOI:** 10.3389/fpsyg.2021.623668

**Published:** 2021-04-09

**Authors:** Jessica Woodhams, Juliane A. Kloess, Brendan Jose, Catherine E. Hamilton-Giachritsis

**Affiliations:** ^1^Centre for Applied Psychology, School of Psychology, University of Birmingham, Birmingham, United Kingdom; ^2^WMG, The University of Warwick, Warwick, United Kingdom; ^3^Department of Psychology, University of Bath, Bath, United Kingdom

**Keywords:** online (Internet) child sexual abuse, Internet sex offending, offender characteristics, TOR, dark web

## Abstract

International law enforcement have noted a rise in the use of the Dark Web to facilitate and commit sexual offenses against children, both prior to and since the start of the COVID-19 pandemic. The study presented here therefore aimed to investigate the characteristics and behaviors of anonymous users of Dark Web platforms who were suspected of engaging in the sexual abuse of children. Naturally-occurring data on 53 anonymous suspects, who were active on the Dark Web and had come to police attention in the United Kingdom (UK), were sampled. Analysis of the data yielded 462 features that could be coded reliably. Analysis of these features provided novel insights into suspects’ characteristics, their motivations for using the Dark Web, the nature of the offending behavior they reported engaging in, their technical and security precautions, sexual interests, and the content of their interactions with one another. Findings are discussed in relation to theoretical and practical implications, as well as directions for future research.

## Introduction

Internet technologies have been purported to serve various functions for individuals with a sexual interest in children and/or those who engage in the sexual exploitation and abuse of children (hereafter CSEA^1^). Online communities that are geared toward users with an interest in CSEA provide access to: (i) material depicting child sexual abuse (Quayle and Taylor, 2001); (ii) an opportunity to communicate with like-minded individuals (Durkin, 1997; Europol, 2014); (iii) a sense of belonging and acceptance (Quayle and Taylor, 2002; Holt et al., 2010); and (iv) an environment in which sexual fantasies can be shared, verified and gratified (Davidson and Gottschalk, 2011). The latter is very reinforcing given that CSEA is self-/other-justified (Quayle et al., 2000; Quayle and Taylor, 2003; Holt et al., 2010). Clearly, expressing such thoughts and behaviors would be highly stigmatizing in society outside of such communities.

Online communities of individuals with a sexual interest in children have existed on the Surface Web for some time and several reviews of the literature regarding the characteristics of online child sex offenders have been conducted (e.g., Babchishin et al., 2011; Seto, 2013; Kloess et al., 2014; Ly et al., 2018). For example, in a meta-analysis by Babchishin et al. (2011), the authors compared studies on online sexual offenders (who used the Internet to facilitate their offending) to those on offline offenders (who committed contact sexual offenses), and found that online offenders were more likely to be younger and of an ethnic minority. They were also found to be less likely to have a history of physical abuse, report fewer cognitive distortions, including less emotional congruence with children, and present with less socially desirable responding. Compared to offline offenders, they scored higher on sexual deviance and victim empathy. When compared to the wider population, they were more likely to report having experienced physical and sexual abuse, and being unemployed, as well as less likely to be, or have been, married.

More recently, international law enforcement has recorded a rise in the use of the Dark Web by individuals for the purpose of engaging with the topic of CSEA and related material (Europol, 2014, 2016, 2020; National Crime Agency, 2018). The Dark Web refers to “content on the World Wide Web that is not indexed by standard search engines” (Weimann, 2016, p. 175). In 2016, Europol reported that both the number of Dark Web forums dedicated to pedophilia and CSEA, and the volume of material being exchanged thereon, had been increasing. This includes both ‘known’ and first-generation (i.e., new) CSEA material. Similarly, Owen and Savage (2015) collected data on the Dark Web over a period of 6 months, and found that ‘abuse sites’ were by far the most popular (represented by 80% of the total number of requests). Since the outbreak of COVID-19, Europol (2020) have seen a further rise in activity on Dark Web forums dedicated to CSEA. Alongside the rise in frequency, the content depicted in CSEA material has become increasingly more extreme and violent (Europol, 2014).

Membership of Dark Web forums dedicated to CSEA is not insignificant. Web-Iq (2018) reported that within seven such forums they indexed, there were more than two million unique user IDs. While some users register on multiple forums, it was estimated that this number equates to between 300,000 and 1 million users across the seven forums. In light of these findings, enhancing law enforcement’s understanding of the threat posed by users on these forums has been identified as a key priority, as has the prioritization of users suspected of engaging in CSEA for the purpose of identifying them and disrupting offending behavior (Europol, 2016). We cannot assume that behavior displayed by users on the Dark Web is the same as behavior displayed by users on the Surface Web – further research into this is therefore needed in order to ensure that law enforcement efforts are underpinned by a relevant evidence base.

While CSEA offenders who utilize Internet communication platforms on the Surface Web as part of their offending behavior have been studied for some time, there is limited knowledge around and understanding of the behavior of CSEA offenders who make use of such platforms on the Dark Web. By the sheer nature of these platforms on the Dark Web, including TOR^2^, they are spaces of the World Wide Web that are not stumbled upon accidentally. For some forums and other restricted areas, vetting of a potential member is required, which involves them having to supply new CSEA material for entry and on an ongoing basis in order to retain membership (Europol, 2014). As a result, one might expect that members of such forums, and/or other restricted areas, are highly motivated to offend, given the effort expended to access them. Restricted areas are associated with discussions and material that are of a more violent and sadistic nature (Europol, 2014), and the Dark Web is home to bulletin boards for niche sexual interests, including CSEA that involves very young children and sadism (Europol, 2020). Individuals who utilize the Dark Web for the various purposes related to CSEA may therefore present with more deviant sexual interests, for which they are less likely to find a suitable outlet on the Surface Web.

Historically, individuals operating on the Dark Web would have required a certain level of technological expertise. However, Internet communication platforms on the Dark Web are becoming increasingly more accessible (Europol, 2016), meaning that less technologically-sophisticated users are now able to join the various online communities available. Balfe et al.’s (2015) review of the precautions employed by Internet child sexual offenders found that the security measures they employed varied depending on their age and level of experience, with those who were younger and less experienced taking fewer precautions. Overall, across the sample, very few offenders adopted security measures, although it should be noted that while the study sampled 11 years of empirical research (from 2000 to 2011), this would now be considered dated. In addition, the offenders in the samples from the studies that were included in the review had all been apprehended, which is likely to have occurred as a result of their low level of technical sophistication, and the precautions they took (if any). Our paper, in part, responds to the call by Balfe et al. (2015) for further research on the precautions taken by online CSEA offenders, by studying a recent sample of anonymous online CSEA offenders who used Dark Web platforms, *in situ*, and who were undetected at the time of data collection (i.e., their offline identities had not been resolved).

As well as studying the security precautions taken by online CSEA offenders, researchers have considered the types and nature of interactions between members of these online communities in the Surface Web. Holt et al. (2010) note that such communities are self-policing in that members police one another’s behavior to avoid security breaches. Security measures and avoiding detection are therefore a common discussion topic in such communities on the Surface Web (see also Quayle and Taylor, 2001; Davidson and Martellozzo, 2008; Europol, 2015). Members also offer advice, and share ‘tips’ with one another, on accessing victims (Quayle and Taylor, 2001; Davidson and Martellozzo, 2008; Europol, 2014, 2015; Web-Iq, 2018).

O’Halloran and Quayle’s (2010) study of a support forum for ‘boy lovers’ focused on the justifications used and the excuses given by self-identified pedophiles who were members of the forum (sometimes in response to members who were anti-pedophilia). Justifications focused on denying harm or injury to the child as a result of the abuse, claiming that the abuse actually benefitted the child, and that the child deserved or attracted sexual advances from the adult(s) (i.e., denial of victimhood). While not the primary focus of their study, O’Halloran and Quayle (2010) also reported on the purpose of other communications between members. Like the studies above, these included: (i) policing the forum to prevent infiltration (e.g., by law enforcement), (ii) protecting one another (by giving advice), and (iii) setting ground rules regarding what were and what were not the purposes of the forum. Communications that acknowledged the causation of harm to a child were very rare.

The range of forums available on the Dark Web include those that are similar in focus to the ones included in O’Halloran and Quayle’s (2010) study on the Surface Web. We can therefore expect communities on the Dark Web to display similar behaviors. However, within the Dark Web, there are forums and bulletin boards that are specifically dedicated to CSEA that is of a sadistic nature, which is very different given its explicit focus on causing harm through both physical and psychological means (Europol, 2014, 2020). Here, discussions centered around sexual offenses committed against children would likely be characterized by entitlement, indifference to victims suffering, and a lack of remorse (Beech et al., 2005; Mokros et al., 2011).

In summary, while we can hypothesize what similarities and differences there might be in terms of the characteristics and behaviors of individuals who use the Surface Web or the Dark Web (for the purpose of committing offenses relating to CSEA), to date, there has been no study that has examined in depth what these look like on various platforms on the Dark Web^3^. Our study was therefore exploratory and aimed to document, in detail, the characteristics and behaviors of these suspects. Such a study is needed because existing literature on suspects who operate on CSEA sites on the Dark Web neglect to report their methodology (e.g., Web-Iq, 2018), or their findings are based on surveys of law enforcement professionals’ perceptions of the threat (e.g., Europol, 2015). In addition, much of the previous research on Internet child sexual offenders has derived findings from convicted, and therefore apprehended, offenders [see Seigfried-Spellar’s (2014) study of Internet child pornography consumers for an exception].

Users encountered by law enforcement on Dark Web platforms are anonymous. As such, their offline identities are not (yet) known, and they have yet to be apprehended. While sampling apprehended offenders makes sense when using research findings to inform risk assessment procedures and psychological interventions, it is more problematic to use such samples to inform the policing of offenders who have not yet been identified. This is because their characteristics and behavior may be related to the reason(s) for their apprehension, and as such differ in important ways to those who remain unapprehended (e.g., Bennell and Canter, 2002; Neutze et al., 2012). For example, apprehended child sexual offenders may show greater stability in their offending behavior and patterns than those who are unapprehended; this would be similar to findings that unapprehended serial rapists have higher levels of polymorphism than those observed in apprehended serial rapists (Lovell et al., 2017). In addition, research on unapprehended child sexual offenders has found that they differ from those who have been apprehended in terms of their level of sexual preoccupation, breadth of paraphilic interests, coping styles, sexual abuse victimization, education, as well as employment (Neutze et al., 2012). When law enforcement personnel are interacting with suspects of CSEA online, whether assuming the identity of a child or an adult (Wortley and Smallbone, 2012; Martellozzo, 2015), it is important that they are aware of the current knowledge base regarding this group of users, and for this, as well as related research findings, to be incorporated into relevant training.

## Materials and Methods

### Ethics Statement

The research project was granted full ethical approval by the Science Technology Engineering and Mathematics Ethics Committee at the University of Birmingham [ERN_14-1435E], and the Psychology Research Ethics Committee at the University of Bath. Written informed consent from the participants was not required to participate in this study in accordance with national legislation and the institutional requirements. All members of the research team received security clearance to undertake research within a United Kingdom police force. The research project also received scrutiny from the United Kingdom’s Information Commissioner’s Office and the Office for the Surveillance Commissioner, now the Investigatory Powers Commissioner’s Office.

### Sample/Participants

Our sample constituted 53 anonymous individuals who were active on the Dark Web between October 2014 and November 2016, and by virtue of the nature of the platform they were using, had come to the attention of a large United Kingdom police force. Based on the information the individuals had disclosed to others in the Dark Web, they were suspected by the police of committing or having committed a contact sexual offense involving a child and/or an offense relating to CSEA material (i.e., possession, distribution, and production). At the point of data collection, none of the suspects were subject to a formal police investigation. Some became the subject of a formal police investigation at a later stage, but this was not the case for all of them. The 53 suspects were members of and contributed to one of four different TOR forums (geared toward CSEA) that were under surveillance by the United Kingdom police force at the time the research was being conducted (*n* = 27; 51%), alternatively using Internet Relay Chat^4^ (IRC, *n* = 19; 73%), TOR chat (*n* = 6; 23%), or a peer-to-peer file sharing network^5^ (*n* = 1; 4%).

In terms of the nature of the four forums, seventeen suspects were members of a forum which presented children as sexual beings with whom to have a loving relationship, and condoned material depicting the sexual abuse of babies and toddlers (i.e., Forum 1). Eight suspects were members of a second forum, which was the largest forum on the Dark Web at the time of data collection (i.e., Forum 2). Members of Forum 2 varied in terms of sexual interest in age and type of activity, but torture and/or sadistic acts of abuse were not tolerated. One suspect was a member of a forum dedicated to the torture of children and sadism (i.e., Forum 3). A fourth forum, of which one suspect was a member, had varied content but was more focused on torture and sadism (including the killing of children) (i.e., Forum 4). All of the forums were invite-only, including specific criteria that had to be met when a suspect wished to apply for membership.

During data collection, all suspects were anonymous (i.e., their real-world identities were unknown to the police), however, 11 of the suspects’ identities were subsequently resolved and the individuals behind the usernames were charged with having committed sexual offenses against children. Eight of the 11 offenders (73%) were charged with possession of CSEA material, and three (27%) were charged with committing a contact sexual offense against at least one child.

### Procedure

Data were collected on a secure police site. At the time of data collection, the researchers were kept blind to the type of investigation being conducted by the police, and the suspicions regarding the suspect, in order to avoid biasing the coding of data. The data used in the research project consisted of forum^6^ postings and private emails/messages, as well as digital material in the form of images and videos that came from the main forum used by each suspect. These data were derived by means of extracting existing forum postings from networks on the Dark Web for the relevant usernames under investigation. Forum data was limited to the posts of the suspects under investigation rather than also including the posts of other forum members for reasons of privacy and proportionality. These data were not, therefore, interactive. For 26 of the 53 suspects (49%), data from IRC was available; this was interactive and represented interactions between the suspect and an undercover police officer, posing as a suspect. CSEA material (i.e., in the form of both still and moving images) was included in the data of 21 suspects (40% of the sample), where it formed part of the interactions between suspects, or was the subject of forum postings. However, this material is not described further here, as it is not the focus of the analysis presented in the present article.

All of the data available for each suspect were included in the analysis; the data were the original contributions made by suspects, as recorded on the relevant Internet communication platform. The number of pages of available data per suspect varied and ranged from 1 to 12 transcripts (*M* = 4.63, *SD* = 3.66), and 1 to 411 pages (*M* = 25.82, *SD* = 73.76) respectively, resulting in a total of 1265 pages (single-spaced format, font size 12) of data.

### Data Analysis

The transcripts of forum postings and private emails/messages were formatted to enable their importing into MAXQDA12, a professional software package, to facilitate the process of qualitative data analysis. The purpose of the coding was to identify features that described the online activity and behavior of the suspects. As features were identified, they were recorded in the coding scheme as a code using a descriptive label. The textual data was annotated with the relevant code by highlighting the relevant area of text and linking it to the corresponding codes or sub-codes in the coding framework. The coding and qualitative analysis was completed by the second author on a line-by-line basis. An inductive, content-driven approach was taken in order to allow for the identification of any key information, trends, themes or ideas apparent across the data (Guest et al., 2012).

Additional codes were developed as new features were identified, while reappearing elements were assigned codes that already existed within the coding scheme. Codes and sub-codes were grouped in order to form superordinate codes relating to the key concepts under investigation. A hierarchical grouping style facilitated the organization of codes and sub-codes according to their similarity and relationship with each other within these key concepts.

Throughout the analysis, an iterative approach was employed by revisiting and adjusting existing codes and sub-codes, as well as revising them, where appropriate. Additionally, textual data within codes and sub-codes were re-examined to ensure that codes described the information within them accurately. Transcripts were re-read and segments of text re-coded, where necessary. If new information or insight was gained, the coding scheme was modified to further develop and refine it. The descriptions of codes and sub-codes were developed throughout, as well as verified and revised through discussions with the research team.

In order to determine whether the data could be coded reliably using the coding scheme, inter-rater reliability was assessed once an initial coding scheme had been developed. This coding scheme contained 479 variables (codes). All coding was conducted by the second author, with a second researcher coding 10% of the data derived from the first five cases (i.e., 10% of the textual data available for each of the first five cases). This researcher was employed on the research project for the purpose of analyzing CSEA material (which is the focus of another article, and will therefore not be presented here). Both coders were experienced in conducting qualitative data analysis. For the purpose of assessing inter-rater reliability, each coder recorded for each variable whether it was present or absent in the material. The level of agreement between the two coders was evaluated using percentage agreement and Cohen’s Kappa. Percentage agreement is a simple indicator of reliability; in contrast, Kappa is a conservative measure of inter-rater reliability, used to assess the reliability between two coders with categorical or ordinal data, and with the added benefit that it corrects for chance agreement. There can be a Kappa Paradox, where the percent agreement is very high, but the Kappa is very low; this tends to occur when almost all cases fall into one category. These measures of inter-rater reliability were calculated on a case-by-case, as well as variable-by-variable, basis. Table 1 presents the κ-values for the case-wise analysis. All statistics were in the acceptable range, namely ≥80% for percentage agreement (Hartmann, 1977), and ≥0.61 for Kappa (Landis and Koch, 1977).

**TABLE 1:** Inter-rater reliability values across cases.

	**Case 1**	**Case 2**	**Case 3**	**Case 4**	**Case 5**
% agreement	0.96	0.99	0.98	0.99	0.99
Kappa	0.77	0.62	0.79	0.73	0.69

When reliability was assessed on a variable-by-variable basis, 60 of the 479 variables achieved a kappa of 1.00, and percentage agreement of 100%. 377 variables achieved 100% percentage non-occurrence agreement (i.e., both coders agreed that the variable was not present in any of the five cases), and 31 variables achieved 80% percentage non-occurrence agreement. Seven variables achieved a Kappa of less than 0.60 (i.e., 0.55), but did achieve an acceptable level of percentage agreement (i.e., 80%). Only four remaining variables achieved both an unacceptable Kappa value, and a percentage agreement of less than 80%. Inspection of the occasions of coding where there was disagreement revealed no clear pattern of disagreement; instead, they were genuine coding errors on the part of the second coder. The initial coding completed by the primary coder (the second author) for these five cases was therefore retained.

Having established inter-rater reliability, the remaining case material was coded by the second author. As is to be expected with data of this nature, the coding scheme continued to be refined, and expanded upon, as new information became apparent, and new features were identified. This resulted in a final coding scheme of 462 variables (codes). The development of the coding scheme, and the coding of the 53 cases, took the duration of the research project (i.e., 18 months). The length of time taken per case was highly variable, and depended on the amount of data available per case.

## Results

A wealth of information was identified in the textual data. While our main focus lay with the behaviors and characteristics displayed by suspects as part of their online communication/interactions, a range of features were coded. These related to (i) the suspects’ demographics (e.g., gender, relationship status, and employment); (ii) their self-reported motivations for and use of various Internet communication platforms (e.g., accessing CSEA material, chatting, forums, and P2P file sharing network); (iii) security measures and precautions suspects reported taking (e.g., not using a webcam/not showing their face); (iv) their self-reported sexual interests and likes; (v) their behaviors on the relevant Internet communication platform (e.g., discussing/sharing *modus operandi*, encouraging other users to contribute to the forum and share material, requesting information and/or material); as well as (vi) the various topics contained within posts on the forum (ranging from advice about how to approach and interact with children to how to avoid detection, to various different materials related to CSEA). Key findings are reported below under common themes. Unless otherwise stated, percentages cited are a proportion of the overall sample of 53 suspects.

### Suspects’ Demographic Characteristics

Based on suspects’ self-reported gender, all except one were male. The exception refers to what appeared to be a female suspect – this assessment was based on their username, and the type of offending behavior and descriptions of abuse reported by them. As defined on the forum, 13% (*n* = 7) of suspects self-reported holding a senior position on the main forum (from which their data was derived) (i.e., owner, administrator or moderator). 28% (*n* = 15) of the suspects reported having biological children of their own. Two of these suspects (13%) also reported having access to other children, and a further three suspects (6%, who did not disclose having biological children) reported generally having access to children (e.g., extended family members). In total, therefore, 34% (*n* = 18) of our sample reported having regular access to children.

### Technical and Precautionary Behavior

As part of their forum posts, or during conversations/interactions with others, suspects explained why they were using the platforms they did. For some, it was accessing CSEA material (32%; *n* = 17), and for others, it was conversing with like-minded users (23%; *n* = 12). Eight suspects (14%) self-reported producing CSEA material, and 11 suspects (21%) self-reported being in possession of CSEA material.

The suspects took a range of precautions in order to avoid detection, and to maintain their anonymity. These included: (i) cautioning one another about their behavior (23%; *n* = 12); (ii) keeping personal information concealed (e.g., face, identifiers, location) (9%; *n* = 5); (iii) informing one another about legislation (6%; *n* = 3); (iv) informing one another about major legal cases; (v) using encryption software and TOR (9%; *n* = 5); and (vi) not distributing material (11%; *n* = 6). The majority of suspects’ usernames (89%, *n* = 47) were not related to a sexual interest in children/an interest in CSEA.

### Reported Engagement in Child Sexual Abuse

The 53 suspects in our sample self-reported engaging in a range of sexual behaviors against children. For 76% (*n* = 40) of the suspects, there was evidence of the possession and collection of CSEA material. For 21% (*n* = 11), there was evidence that they had or were inciting another person to sexually abuse a child. Nine percent (*n* = 5) of the suspects self-reported sexually abusing a child via Internet technologies (e.g., by means of Internet communication platforms, including a webcam). For 64% (*n* = 34), there was evidence that they had or were engaging in the sexual abuse of a child in person (i.e., committing a contact sexual offense). While none of the offending behavior engaged in by the sample of 53 suspects resulted in the death of a child, at least for some, their described actions reportedly involved the physical abuse of a child (15%; *n* = 8), or clearly fulfilled the function of a sadistic sexual interest (13%; *n* = 7).

In terms of cross-over of offending behaviors, for 8% (*n* = 4) of the 53 suspects, there was evidence that they were sexually exploiting and abusing children both online and in the physical world. For 43% (*n* = 23) of suspects, there was evidence that they were in possession of and/or collected CSEA material, as well as committed sexual offenses against children in the physical world.

### Topics Posted

We analyzed the nature of posts made by suspects on the relevant forums to which they belonged. This is presented as a separate subsection from our other results because posting to a forum is less interactive than conversing with other offenders, with some suspects solely engaging in posting behavior rather than interacting with other users. Thus, it was important to capture content across all the suspects.

A broad range of topics were identified, some of which were merely mentioned by a few suspects (i.e., 2–10%), while others were more common. These were predominantly related to sexual acts (40%; *n* = 21) (including various forms of penetration, such as anal, oral, and digital), and deviant sexual interests (38%; *n* = 20), such as bestiality, breast-feeding, incest, female genital mutilation, feces and sadism, with the latter being one of the most prevalent (i.e., 30%, *n* = 16). Some of the posts were about a particular age group of victims (e.g., babies [15%; *n* = 8]). Issues related to security measures and precautions, with a view to avoiding detection, were also frequently posted, including updates on law enforcement activity (23%; *n* = 12). Posts also related to discussions that contrasted individuals with sexual interests in children of different ages (11%; *n* = 6), as well as predilections for inflicting pain vs. sexual abuse (6%; *n* = 3). Finally, some posts related to the rules of the group and expected conduct, including topics that were out-of-bounds (21%; *n* = 11).

### Sexual Interests

Suspects were recorded as presenting with a sexual interest in a particular topic or subject matter if they self-disclosed it as part of forum postings, conversations or interactions, or where they (re-) posted content/material in a manner that was endorsing rather than critical of it. Whether they were sexually interested in a topic was clear from the statements and/or postings they made (e.g., “I find X a real turn-on”). Of the 53 suspects, 19 (36%) self-disclosed a liking/interest in one or more different types of sexual interest. Sadism (26%; *n* = 14) and incest (17%; *n* = 9) were the sexual interests self-reported by most suspects. The other sexual interests self-reported by suspects in order of frequency were: bestiality (8%; *n* = 4), urination (8%; *n* = 4), nappies/diapers (6%; *n* = 3), dressing-up (6%; *n* = 3), child dolls (6%; *n* = 3), and feet (4%; *n* = 2). All references to a sexual interest in sadism and urination were specifically related to children (as opposed to adults). References to incest were clearly in relation to children for four suspects, however, this could not be conclusively verified for the remaining five suspects (although it very likely related to pre-pubescent children due to the nature of the forum in which the material was posted). In terms of the association between the remaining sexual interests and children (as opposed to adults, or this being unspecified), the representation was as follows: dressing up (4%; *n* = 2), nappies (4%; *n* = 2), feet (2%; *n* = 1), and bestiality (2%; *n* = 1).

Where disclosures of sexual interests were made by suspects (*n* = 19), the number of different types self-reported across suspects ranged from one to six: one (47%; *n* = 9), two (26%; *n* = 5), three (2%; *n* = 1), four (4%; *n* = 2) and six (4%; *n* = 2). While it would have been interesting to examine in more detail any associations between a suspect’s type of sexual interest, as well as the gender and age (child and/or adult and/or stage of sexual development) of the focus of their sexual interest, there were insufficient cases to facilitate this.

For 36 suspects, we were able to record their sexual interests in terms of gender, age, and/or stage of sexual development. Clearly, all 53 suspects were operating on Internet communication platforms that were geared toward users with an interest in CSEA, and as such it is unsurprising that 35 of the 36 suspects (97%) specifically disclosed a sexual interest in children. Of these 35, four (11%) also self-reported having a sexual interest in adults. A further one suspect (2%) specifically disclosed a sexual interest in adults only. For the remaining 17 suspects (32%), there was no explicit disclosure of sexual interest relating to age.

In terms of sexual interests around children’s gender, seven of the 35 suspects (19%) disclosed a sexual interest in children of both genders, seven (19%) disclosed a sexual interest in female children, and six (17%) disclosed a sexual interest in male children. The remaining 15 out of the 35 suspects (43%) who disclosed a sexual interest in children did not specify any particular interest in terms of the gender of a child. Twenty-nine suspects expressed a sexual interest related to the stage of sexual development of a child. Ten suspects (34%) expressed a sexual interest in children under 5 years of age. The reference to ‘under 5 years’ is not a category created by the researchers, but is used by the suspects themselves to specifically refer to children under the age of 5 years. Thirteen (45%) expressed a sexual interest in pre-pubescent children, nine (31%) in early-pubescent children, and four (14%) in pubescent children. ‘Pre-pubescent’ refers to children whose body shows no sign of any development of secondary sex characteristics; ‘early-pubescent’ refers to children whose body shows initial development of secondary sex characteristics; ‘pubescent’ refers to children whose body shows development of secondary sex characteristics.

Regarding polymorphism (i.e., cross-over in sexual interest), four of the 36 suspects’ (11%) self-disclosures implied this for age (i.e., child/adult), seven for gender of child (19%), and five suspects self-disclosed a sexual interest in children spanning different developmental stages (14%), predominantly the two youngest categories (i.e., under 5 and pre-pubescent). In the under-5 years and pre-pubescent categories, there was a mix of suspects sexually interested in females, males and also both genders (where gender was specified by the suspect).

### Suspect-Suspect Interaction Behaviors

The way in which the suspects interacted with one another was varied. Given the nature and focus of the forums (from where most data were derived), it is unsurprising that the topic of CSEA featured in most of their interactions. This went beyond merely reporting engagement in CSEA; for example, suspects discussed their experiences of committing sexual offenses against children (15%; *n* = 8), their own victimization as children (6%; *n* = 3), and the sharing of their biological children for sexual abuse by others (4%; *n* = 2). They exchanged CSEA material (6%; *n* = 3), and engaged in promoting and selling it (2%; *n* = 1).

Communication between suspects, however, was not solely related to CSEA, but included general chat and conversation-making, as well as sexually explicit chat. The occurrence of general chat was more frequent in Dark Web communities than on IRC. Forensic linguistic research recognizes the process of the creation of communities of practice, including in Dark Web forums (Grant and MacLeod, 2018; Chiang, 2019). Within our dataset, group identity was apparent through the explicit welcoming of new members (*n* = 11). In addition, suspects supported each other by offering advice and suggestions around (i) ‘finding’ victims and particular material; (ii) *modus operandi*; (iii) security measures and precautions (e.g., safeness of payment services and software programs) in order to avoid detection; and (iv) how to interact and behave both online and offline if presenting with a sexual interest in children. As far as we could determine, there appeared to be different motivations for such demonstrations of expertise, with some suspects engaging in posturing and attempting to show superiority. For others, it appeared to be shared interests, and the potential for creating new material.

Acts of dominance were also exhibited, whereby some suspects challenged the behavior and contributions of others. This included challenging the correctness of information posted (8%; *n* = 4), the quality of material shared (with regard to the material’s realness, newness and content in terms of the age of the child depicted) (26%; *n* = 14), the feasibility of the abuse claimed to have been perpetrated (11%; *n* = 6), as well as making accusations that the material had been stolen (4%; *n* = 2). In addition, suspects expressed disapproval (26%; *n* = 14), disappointment (15%; *n* = 8), and dislike of one another (4%; *n* = 2). On being challenged, some suspects responded by apologizing (13%; *n* = 7). Positive endorsements of one another’s behavior were also seen in the form of (i) thanking others for positive comments (8%; *n* = 4), (ii) sharing material (26%; *n* = 14), (iii) responding positively to material (74%; *n* = 39), and (iv) re-posting posts (13%; *n* = 7).

In our sample of 53 suspects, five (9%) suggested arranging a physical meeting with another suspect, and two (4%) offered their contact details to other users. As might be expected, there was a relatively high occurrence of statements made by suspects that sought to (i) normalize and minimize the physical and psychological harm suffered by children in the context of CSEA (34%; *n* = 18); (ii) support and advocate for child sexual abuse (17%; *n* = 9); and (iii) objectify children (i.e., referring to children as existing to serve the sexual needs of others, and being ‘deserving’ of abuse) (30%; *n* = 16).

## Discussion

Given the increasing use of the Dark Web by individuals to engage with CSEA material, and interact with like-minded users on forums that are dedicated to CSEA, it is important to establish an evidence base on how such individuals operate on Dark Web platforms. Our study is novel in terms of its in-depth examination of the characteristics and behaviors of 53 suspects of CSEA who used a number of different Dark Web platforms for various purposes related to CSEA. Furthermore, by studying unapprehended suspects and their behaviors *in situ*, through the use of naturally-occurring data, the present article makes an original contribution to the literature on Internet child sexual offending in general, and responds to calls for more research of unapprehended suspects in particular (O’Halloran and Quayle, 2010).

The dataset comprised 53 cases identified from various Internet communication platforms on the Dark Web, which were active at the time of the study, providing conversational data in the form of forum postings, chat and private messages that were derived from interactions between suspects and with undercover police officers. For those suspects who were members of forums, they held positions of varying authority (i.e., some were owners, and some were administrators and moderators). They also varied in how much they contributed to the forum of which they were a member.

Regarding our findings of self-disclosed demographic characteristics, like most studies of perpetrators of online CSEA, whether apprehended or unapprehended (Beier et al., 2009; Aslan and Edelmann, 2014), the vast majority of our suspects were male. 34% reported having access to children who were their own/biological children or extended family members. This compares to Long et al.’s (2016) figures of 42%, and 46% respectively, Beier et al.’s (2009) figures of 28–37%, and Shelton et al.’s (2016) figure of 27%, in terms of offenders having access to and sexually abusing their own children.

Many studies of online child sex offenders have focused on individuals convicted or charged with possession and distribution of CSEA material (i.e., indecent images of children, sometimes known as “child pornography”), with a smaller number including within their sample offenders who engaged in the sexual grooming of children (Babchishin et al., 2011). However, online child sex offending behavior is broader than this (Clevenger et al., 2016), and this breadth of behavior was evident in our Dark Web sample. The majority of our sample were in possession and/or collected CSEA material. In addition, most suspects reported that they were (or had previously) engaged in the sexual abuse of a child in the physical world (i.e., a contact sexual offense). With the advent of digital devices and computers, it is also possible for offenders to commit very serious sexual offenses against a child via Internet technologies (including a webcam and recording facilities; Whittle et al., 2013a; Kloess et al., 2015), which have been found to be just as harmful as a traditional, offline contact sexual offense with regard to mental health outcomes and subsequent suicidal behavior (Hamilton-Giachritsis et al., 2017, 2020). As such, it was important to capture forms of CSEA that have previously been neglected by historic papers, or (inadvertently) trivialized through the use of dichotomies of ‘contact-driven’ and ‘fantasy-driven’ to describe offenders (Briggs et al., 2011). In our sample, a small number of suspects self-reported sexually abusing a child via webcam, and one-fifth were inciting others to sexually abuse a child.

The reasons suspects revealed for using the Dark Web echo the assertions by Durkin (1997), and those reported in studies of other child sexual offenders (e.g., Shelton et al., 2016), with regard to using the Internet for purposes related to CSEA: (i) to access CSEA material, (ii) to converse with like-minded individuals, and (iii) to share self-generated material of CSEA with others. The efforts required from the suspects in our sample in terms of accessing the relevant forums, or in obtaining material from other suspects, gives an indication of their motivation to engage in this type of offending behavior.

Risk assessment and risk management by suspects is critical to their ‘safe’ use of the Internet to facilitate their engagement in CSEA, whether that be the ‘grooming’ of children for CSEA (Whittle et al., 2013b; Kloess et al., 2015; Kloess et al., 2019), or maintaining the security of an online community (Holt et al., 2010; O’Halloran and Quayle, 2010). Risk management and security measures/precautions were also a notable focus in the suspects’ communications we sampled. Advice and discussions centered around maintaining anonymity by concealing personal identifiers (e.g., face and name), rules of conduct within the online community, what to say to victims to avoid detection by others, advice on technical means of avoiding detection (e.g., use of encryption software or TOR), not distributing CSEA material, and updating one another on legislation or new major police/legal cases. These topics relate to precautionary features of models of ‘grooming,’ particularly overcoming external inhibitors (Finkelhor, 1984), grooming the environment and others (Craven et al., 2006), and preventing discovery (Sullivan, 2009).

Despite existing studies citing the employment of usernames associated with CSEA by online child sexual offenders (O’Connell, 2003), the majority of suspects’ usernames in our sample were not related to a sexual interest in children/CSEA more broadly. Whether these differences between studies stem from variations in sample composition (e.g., heightened security awareness in our sample given their use of the Dark Web), or a change over time in choices regarding usernames (possibly in response to law enforcement activity), is unclear.

As noted, a small minority of suspects were explicit about having a sexual interest in adults, in addition to a sexual interest in children. Where it was stated, the number of suspects with a sexual interest in one gender of children, versus a sexual interest in both genders, was comparable. This contrasts with Neutze et al. (2012), where a larger proportion of the sample showed a gender preference (i.e., 94–96%); however, it should be noted that self-disclosures about gender-related sexual interests regarding children were not made by more than half of the sample and, as such, it is unknown whether they had any.

Within our sample, some suspects self-disclosed specific sexual interests in and preference for types of CSEA material in their discussions and postings (e.g., “I don’t mind boys or girls, but girls under 5 really do it for me”). Most of these suspects merely self-disclosed one sexual interest, while other suspects self-disclosed a sexual interest in a wide range of types (e.g., sadism, incest, bestiality, defecation, and urination). The types of CSEA material most frequently mentioned by suspects were sadism and incest. This echoes reports from Europol (2014, 2020) of the Dark Web as an enabler for those with sadistic and other niche sexual interests. In line with Lanning (2012), there was a subset of suspects who presented with diverse sexual interests, whether this was in terms of the nature of the material or the type of victim they expressed an interest in. Lanning (2010) also reported a relationship between developmental stage/age and gender of victim, whereby the older and more sexually developed the child, the more likely it was that the offender had a strong preference for gender^7^. While our data suggest that those who disclose no preference in terms of gender do express a sexual interest in younger, pre-pubescent children, some of the suspects did not fit this pattern.

Suspect-to-suspect behaviors displayed as part of their online conversations and interactions, as well as topic postings, gave a clear impression of a community of individuals. New members were welcomed, and there was general conversation-making alongside discussions of CSEA. There were examples of cooperative behaviors, such as providing assistance and expert advice, as well as problem solving. Much like in other online communities, posts were ‘liked,’ and assistance or sharing was acknowledged through thanking. There also appeared to be examples of dominance being enacted through speech acts such as directives (Leech, 1983), which varied in their directness from suggestions around the next stage of abuse against a specific child to giving orders to others. The use of a similar range of directives has been seen within interactions of groups who engage in sexual offending in the physical world (Woodhams et al., 2012). Other acts of dominance included challenging and/or disapproving of other others and their actions.

Within the textual data, there was evidence of suspects making pro-child sexual abuse statements, normalizing and minimizing the harm caused to children as a result of sexual abuse experiences, as well as referring to children as sexual beings. These assertions about the sexual abuse of children are similar to those observed by O’Halloran and Quayle (2010) in their study of interactions among users on a ‘boy-lover’ website on the Surface Web. In addition, there were almost as many suspects who referred to children as objects whose purpose was to serve the sexual needs of others, and, in some cases, as being ‘deserving’ of the abuse. Such statements are not surprising given the sadistic nature of some of the content presented within the forums, as well as the sexual interests of some of the suspects in our sample, who are likely characterized by entitlement, and a lack of empathy and remorse (Beech et al., 2005; Mokros et al., 2011). These assertions and justifications will be discussed in more detail as part of a separate article.

### Limitations

While this paper makes an original contribution to the literature on Internet child sexual offending, and the less well-known features of those suspects who operate on platforms on the Dark Web, it has a number of limitations that need to be highlighted in order to inform the design of future research studies. While an advantage of our data is that they were naturally-occurring, one limitation is that we merely have coded information for suspects/cases where the relevant features were present. There is therefore no way of obtaining a complete dataset for all suspects/cases across the entire coding scheme. Analyses of some features (e.g., sexual interests) were therefore based on a small number of suspects/cases, and should be interpreted with caution. By sheer nature of using naturally-occurring data, our data are less likely to be distorted by impression management that is often observed in qualitative interviews with apprehended offenders, whose future outcomes are likely affected by the way they present themselves. However, it is also important to note that impression management is very relevant within Dark Web communities, where it would influence one’s standing within the group, and the likelihood of receiving new material from other members. Our dataset comprised of a small sample of 53 individuals who had come to the attention of law enforcement due to intelligence that suggested that they were engaging in the sexual abuse of children. It is therefore unlikely that our sample is representative of all suspects and/or users who present with a sexual interest in children, and are operating on platforms on the Dark Web. This selection criterion may well have introduced bias into our findings, and would therefore benefit from further exploration as part of future empirical research. It is important for such research studies to assess the generalizability of our findings with other samples, including those derived from platforms with a different focus. Finally, inter-rater reliability was assessed using 10% from each of the first five cases included in the dataset, once the coding of these had been completed. This was to ensure that the coding scheme could be used reliably before coding was continued. Inter-rater reliability was therefore assessed early on in the analytic process, rather than once all cases had been analyzed, with the coding scheme evolving and being further developed by the second author as they progressed through the analysis of the remaining cases.

## Conclusion

The use of the Dark Web for purposes related to CSEA is growing; the study presented here is therefore timely in its investigation of the characteristics and behaviors of a subset of individuals who operate on platforms on the Dark Web that are geared toward CSEA. Our findings are relevant to law enforcement efforts in combating such offending behavior (e.g., by informing their training and methods of investigation), providing the first description of a subset of individuals, more of whom will be entering correctional and rehabilitative services, and coming onto caseloads of forensic psychologists and other practitioners in the future.

## Data Availability Statement

The datasets generated for this study are not readily available because the data are owned by the police force who collaborated on the study. Any access to the data would require their explicit permission. Requests to access the datasets should be directed to JW, j.woodhams@bham.ac.uk.

## Ethics Statement

The research project was granted full ethical approval by the Science Technology Engineering and Mathematics Ethics Committee at the University of Birmingham (ERN_14-1435E) and the Psychology Research Ethics Committee at the University of Bath. Written informed consent from the participants was not required to participate in this study in accordance with national legislation and the institutional requirements.

## Author Contributions

JW and CH-G designed the original study. JW, CH-G, and BJ sought and secured multiple funding streams for the project. JW and CH-G sought and obtained ethical approval for the study. They supervised the researchers employed on the project (including JK). JK was the primary developer of the coding scheme which forms the basis of the results. The development of the coding scheme was also informed by and refined following contributions from CH-G and JW. JK conducted the coding of the 53 cases. BJ conducted research to provide outcome data for the study. JW assessed the inter-rater reliability of the coding, and conducted the statistical analyses of the data reported in this article. JK produced the Method section. JW is the primary author of this article. JK and CH-G also contributed to the writing of the present article. BJ reviewed and provided feedback on the article. All authors contributed to the article and approved the submitted version.

## Conflict of Interest

The authors declare that the research was conducted in the absence of any commercial or financial relationships that could be construed as a potential conflict of interest.

## Footnotes

1 A range of terms are used to refer to the sexual exploitation and abuse of children. For the purpose of this paper, we will use the term ‘child sexual exploitation and abuse,’ in line with its usage by WeProtect (n.d.) and the United Kingdom Government (Home Office, 2019).

2 TOR (The Onion Router) is software which enables anonymous communication via a free open network with multiple encryption layers (Huang and Bashir, 2016); use of TOR increases the difficulty of tracing internet usage by an individual.

3 This is not to suggest that suspects who use platforms on the Dark Web are a special type of offender, and we acknowledge that such individuals are likely to use the Surface Web too (e.g., Kokolaki et al., 2020).

4 Internet Relay Chat: IRC was created in 1988 and enables real-time text messaging between internet-connected computers. It is mainly used for group discussion in chat rooms but can also be used for private message exchange between two users (Radware: https://security.radware.com/ddos-knowledge-center/ddospedia/irc-internet-relay-chat/#:~:text=IRC%20(Internet%20Relay%20Chat)%20is,side%20and%20client%2Dside%20commands).

5 Peer-to-peer file sharing is the distribution and sharing of digital media using peer-to-peer (P2P) networking technology. P2P file sharing allows users to access media files, using a P2P software program that searches for other connected computers on a P2P network to locate the desired content.

6 An Internet forum, or message board, is an online discussion site used by users to hold conversations in the form of posted messages. Some forums have an option for exchanging private messages.

7 Lanning’s (2010) publication is based on a database of cases that he has collated during his 27 years in practice. The exact size of the database is unclear with him referring to it as containing “thousands” of cases (p. 3). While he comments on a relationship between gender preference and age of child, no statistical output is provided.
